# Proximal stent migration during coarctation of aorta stenting

**DOI:** 10.1186/s43044-025-00637-z

**Published:** 2025-04-22

**Authors:** Aamir Rashid, Vamiq Rasool, Jan Mohammad, Imran Hafeez, Shahood Ajaz, Hilal Rather

**Affiliations:** Sher-I-Kashmir Institute of Medical Sciences, Srinagar, India

**Keywords:** CoA stenting, Proximal migration, Transcatheter

## Abstract

**Background:**

Transcatheter stenting has become the preferred treatment for native and recurrent coarctation of aorta (CoA), but complications such as stent migration occur in approximately 5% of cases. Proximal stent migration is particularly challenging and often requires surgical intervention. This report highlights the successful transcatheter management of proximal stent migration during CoA stenting in a high-risk patient.

**Case presentation:**

A 22-year-old woman with Turner syndrome and chronic idiopathic thrombocytopenia purpura (ITP) presented with severe native CoA and refractory hypertension. Echocardiography revealed severe left ventricular hypertrophy and bicuspid aortic valve with mild aortic stenosis. The CoA segment gradient was 90 mmHg. During stent implantation using a 16 × 44 mm Zephyr stent mounted on an Atlas balloon, the stent migrated proximally into the right brachiocephalic artery despite appropriate crimping and hypotensive pacing. The stent was stabilized using a pigtail catheter via the right radial artery, and a low-profile peripheral balloon was inflated distal to the stent to pull the system back. However, the stent became stuck at the tightest segment of the CoA. Predilation of the CoA site with a larger balloon widened the segment, allowing the stent to be repositioned and deployed successfully. Post-procedure, the gradient across the CoA decreased to less than 5 mmHg. The patient was discharged after two days without complications, and follow-up imaging confirmed proper stent placement without restenosis.

**Conclusions:**

Our case illustrates the transcatheter management of proximal stent migration during CoA stenting, potentially reducing the need for surgical intervention. A stepwise strategy involving stent stabilization, low-profile balloon-assisted repositioning, and predilation of tight CoA segments can facilitate successful stent repositioning. This case contributes to the existing literature by documenting the occurrence and management of this rare complication.

## Background

Coarctation of the aorta (CoA) accounts for 5–8% of all congenital heart defects. Transcatheter stent implantation has become the standard of care for native or recurrent coarctation [1]. Stent migration is a potential procedure complication observed in approximately 5% of cases [2]. Although proximal stent migration usually requires surgical intervention [3, 4], transcatheter management has been described in few cases [5, 6]. We describe a case of successful transcatheter management of this unusual complication.

## Case presentation

**Fig. 1:**
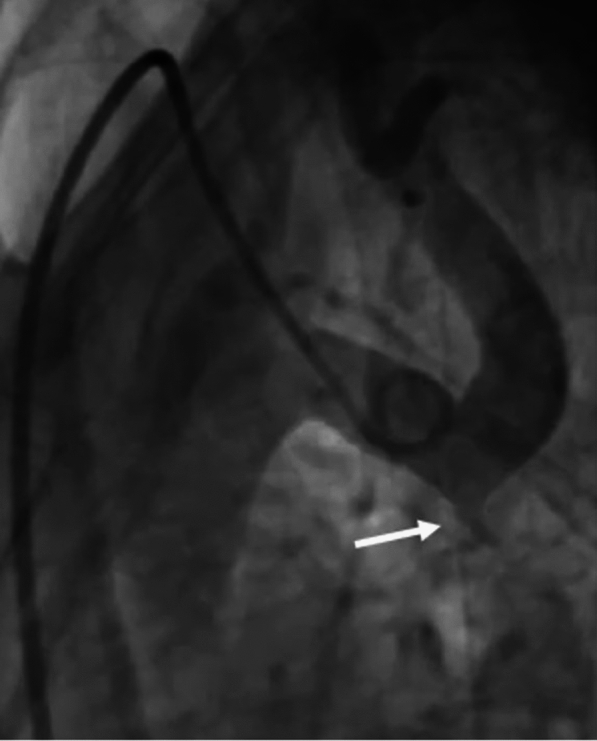
Aortic arch angiogram from right radial route showed tight post-subclavian CoA

A 22-year-old lady was admitted to our cardiology unit with a diagnosis of Severe native CoA for percutaneous stenting. The patient was diagnosed with Turner’s Syndrome with Chronic Idiopathic thrombocytopenia purpura (ITP). She had refractory hypertension despite multiple antihypertensives. Echocardiography showed severe concentric left ventricular hypertrophy with bicuspid aortic valve with mild aortic stenosis. The gradient across the CoA segment was 90 mmg with diastolic tailing. We obtained right femoral artery access using a 5-French micropuncture set under ultrasound guidance. Additionally, we secured 6-French left femoral vein access for placing a temporary pacing lead. A 6-French right radial access was obtained to position a pigtail catheter in the aortic arch for angiographic guidance during stent placement. The initial femoral arterial pressure before the intervention was 80/60 mmHg, while the ascending aortic pressure was 170/70 mmHg, resulting in a gradient of 90 mmHg. The aortic arch angiogram from the right radial approach revealed severe post-subclavian CoA (Fig. 1). The pre-CoA segment measured 18 mm, while the post-CoA dilated segment measured 22 mm. We selected a stent diameter of 16 mm, approximately 80–90% of the pre-CoA segment diameter. A stent length of 40 mm was chosen to adequately cover the CoA segment while minimizing excessive protrusion into adjacent structures.

**Fig. 2:**
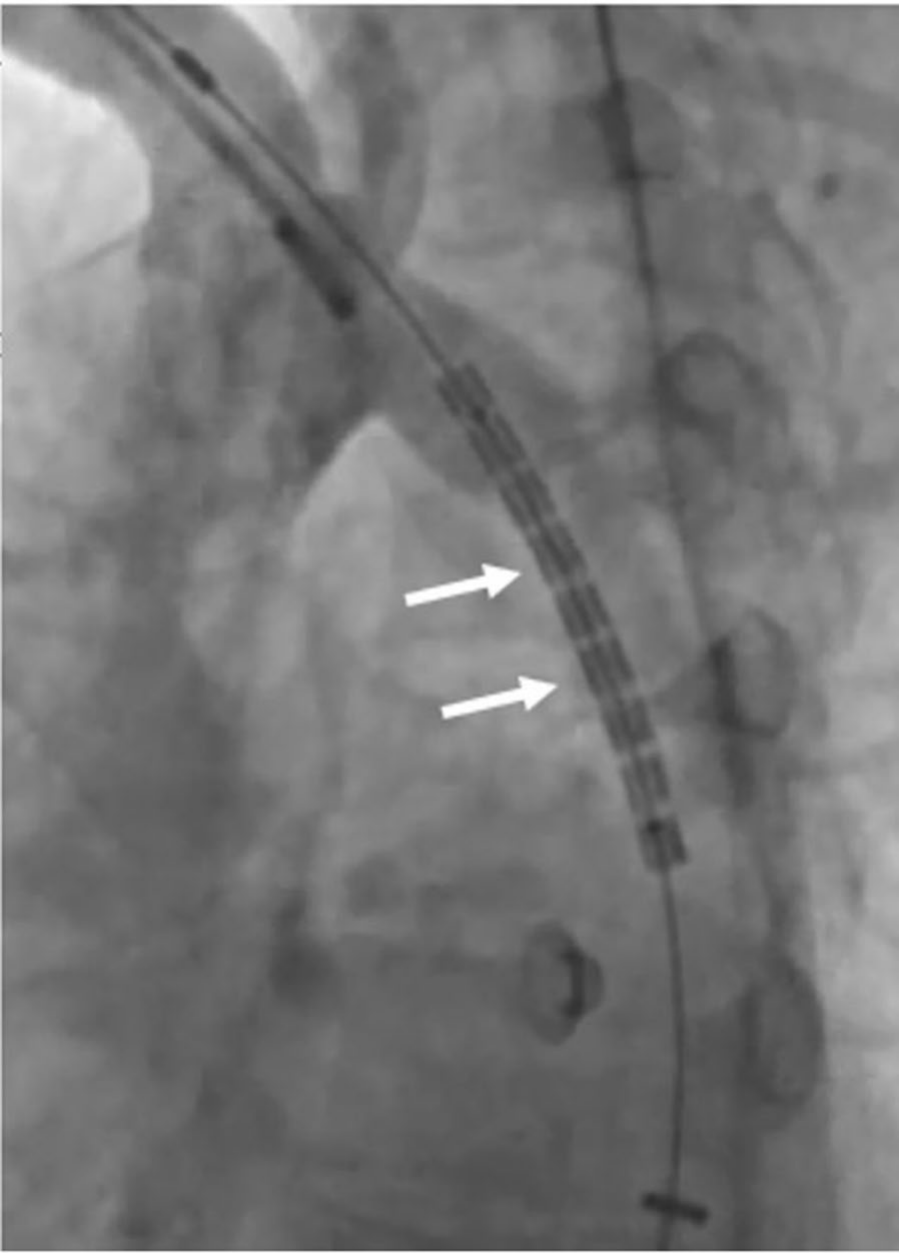
A 16 × 44 mm uncovered stent (Zephyr) mounted on Atlas balloon being positioned across CoA segment

The CoA site was crossed from the femoral route with 0.35 Terumo wire with the Judkin right catheter. The CoA segment measured 3 mm; hence, pre-dilatation was done with an 8 mm × 20 cm peripheral balloon. Typically, balloon diameter for pre-dilatation should not exceed three times the diameter of narrowest segment. After that, a 16 × 44 mm uncovered stent [Zephyr stent (Sahajanand Laser Technology Limited, Gandhinagar, India)] mounted on an Atlas balloon (16 × 40 mm) was positioned across the CoA segment with 14 French Cooks sheath (Fig. 2) with hypotensive pacing rate of 180 beats per minute.

**Fig. 3:**
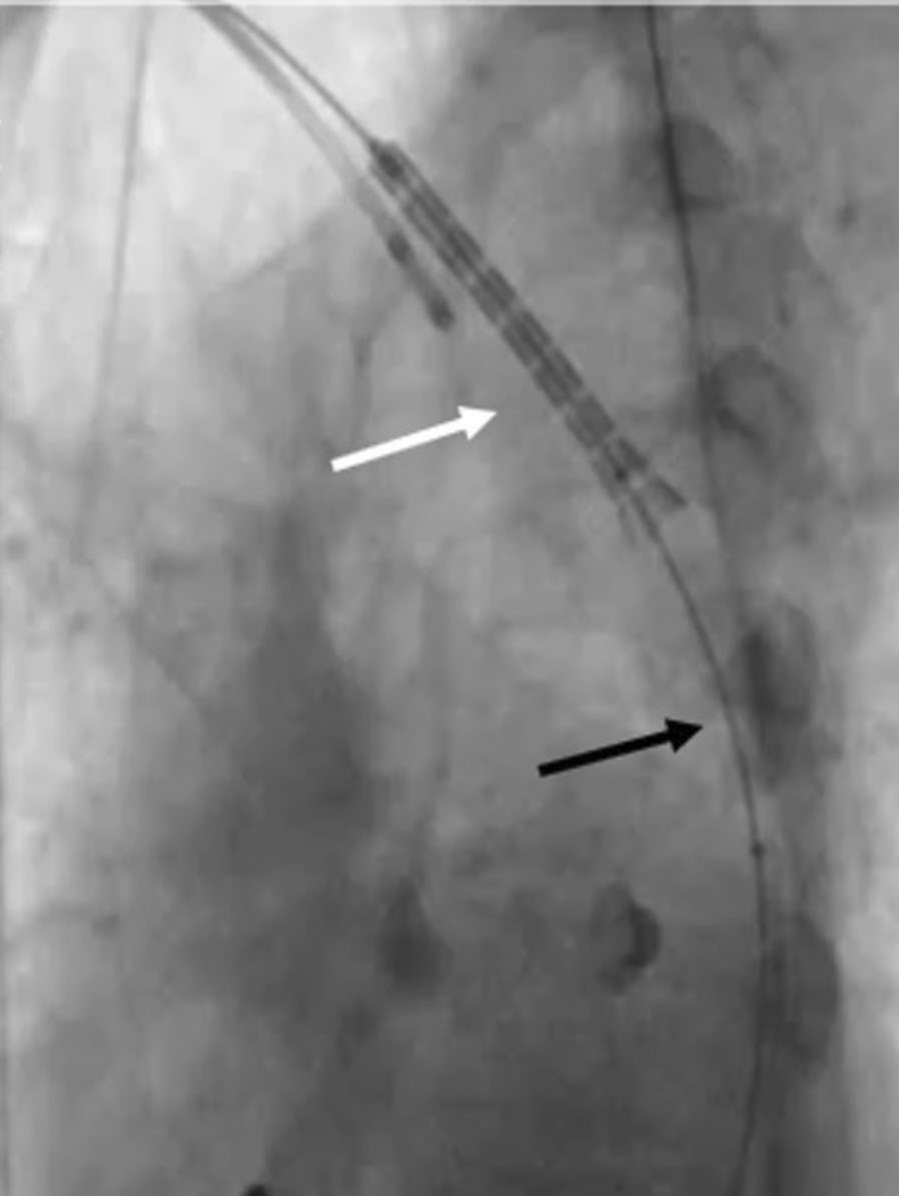
Slippage and proximal migration of the stent into the arch

However, the stent slipped off the balloon during inflation and migrated proximally into the arch (Fig. 3).

**Fig. 4:**
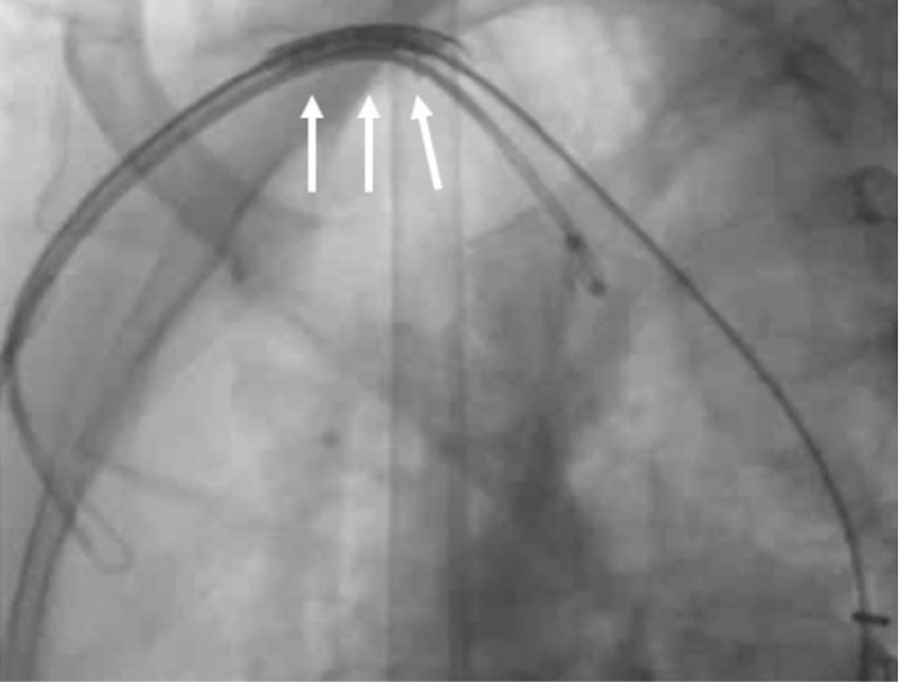
Migration of the stent further into the right brachiocephalic artery

As we tried to manipulate the system, the stent further migrated to the right brachiocephalic artery (Fig. 4).

**Fig. 5:**
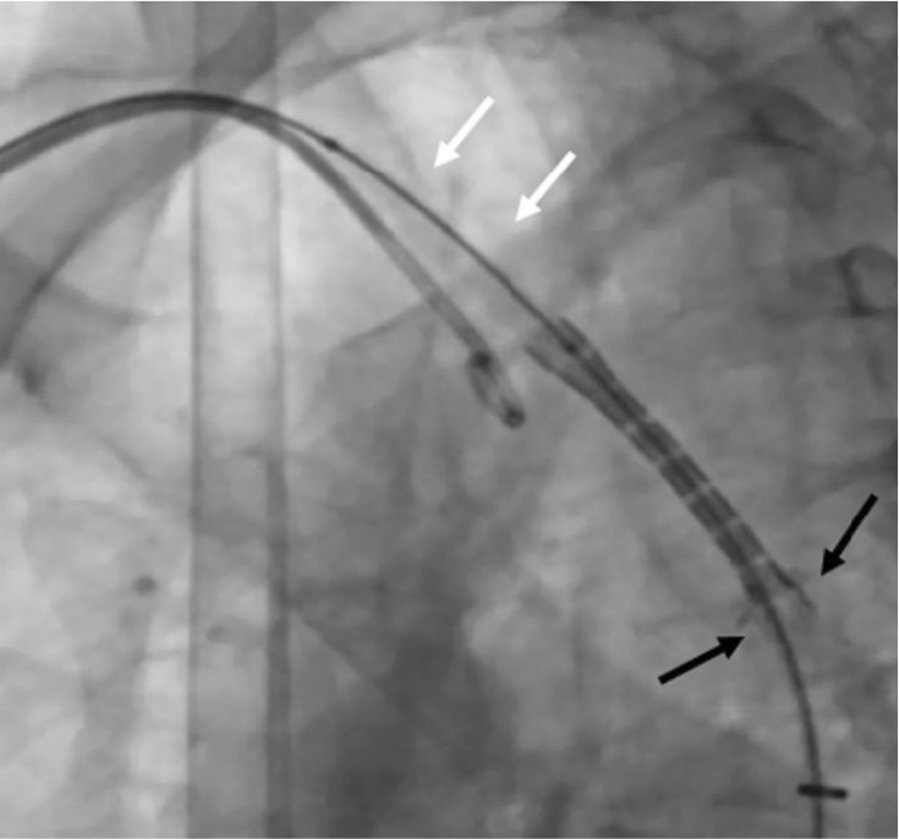
Low-profile balloon (8 mm × 2 cm) distal to stent being inflated and entire system being pulled back

The pigtail through the right radial route helped stabilize it and prevent further migration. We passed a low profile, shorter peripheral balloon (8 mm × 2 cm) through the stent and distal to it, inflated it, and pulled the entire system back (Fig. 5).

**Fig. 6:**
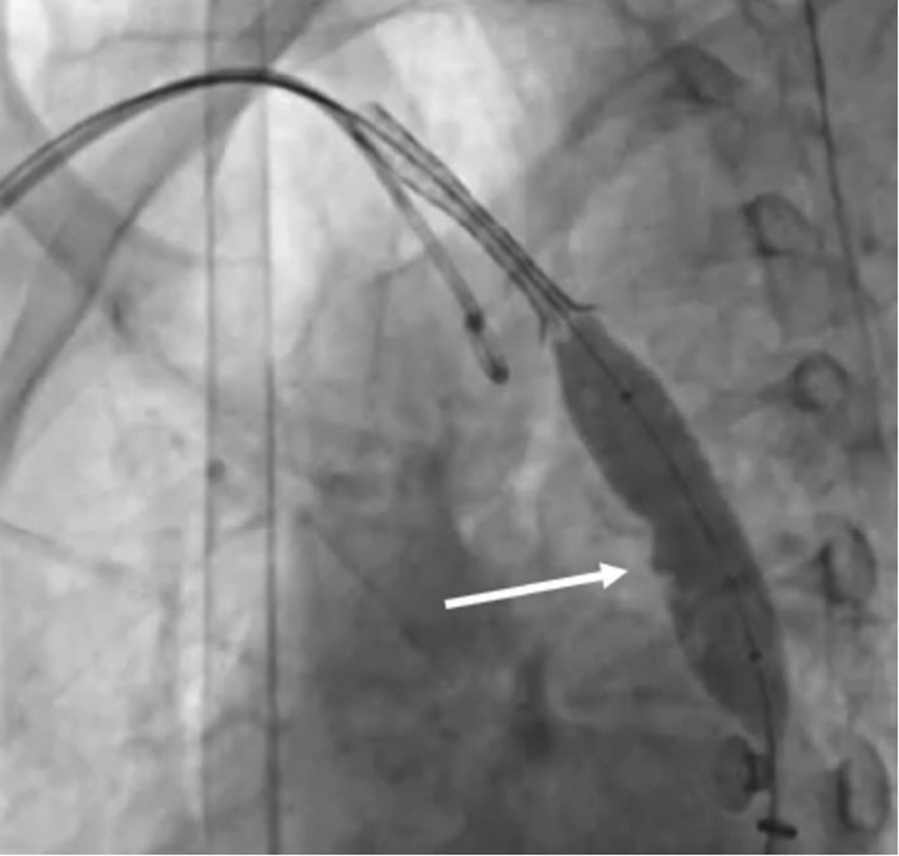
CoA segment being dilated with bigger 12 mm × 4 cm balloon

However, the lower edge of the stent got stuck at the tightest segment and was not coming down despite the pull. To overcome this, we dilated the CoA segment with a bigger 12 mm × 4 cm balloon (Fig. 6).

**Fig. 7:**
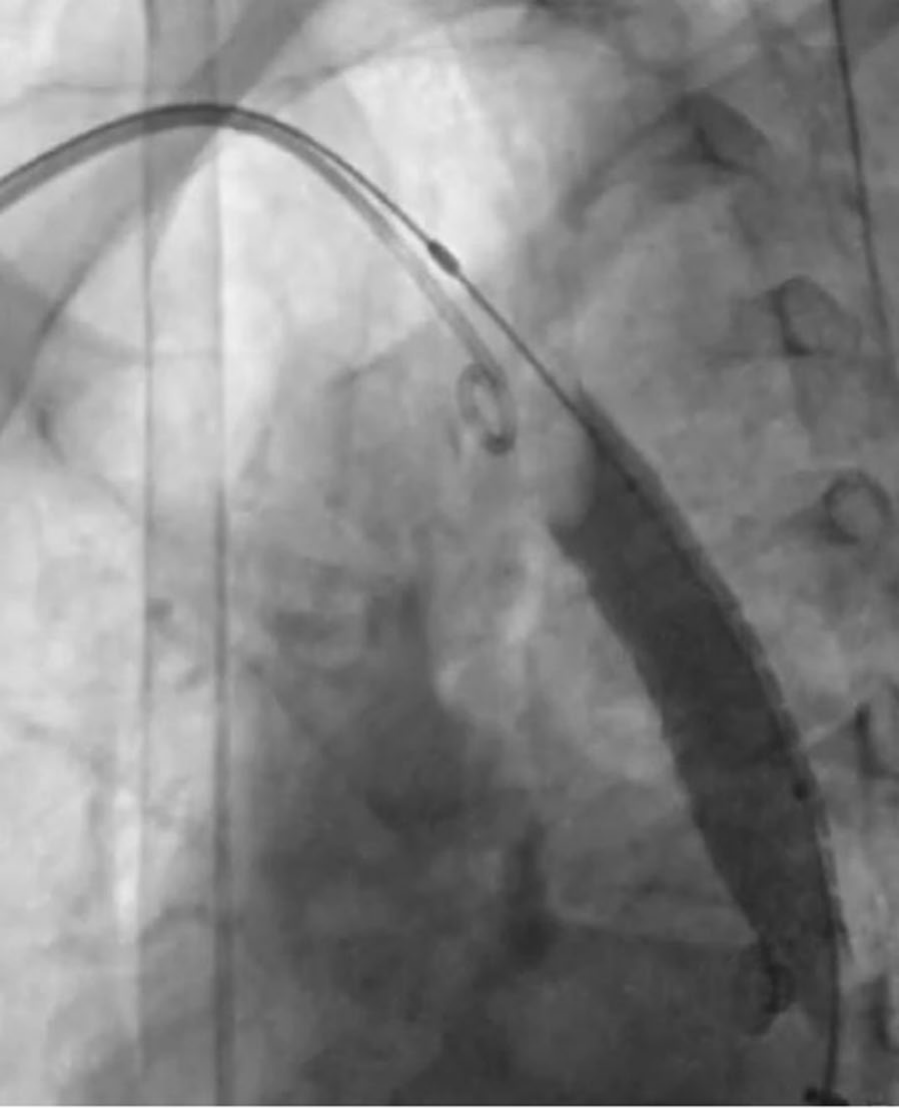
Stent being dilated with larger 16-mm balloon and fully deployed across CoA segment

**Fig. 8:**
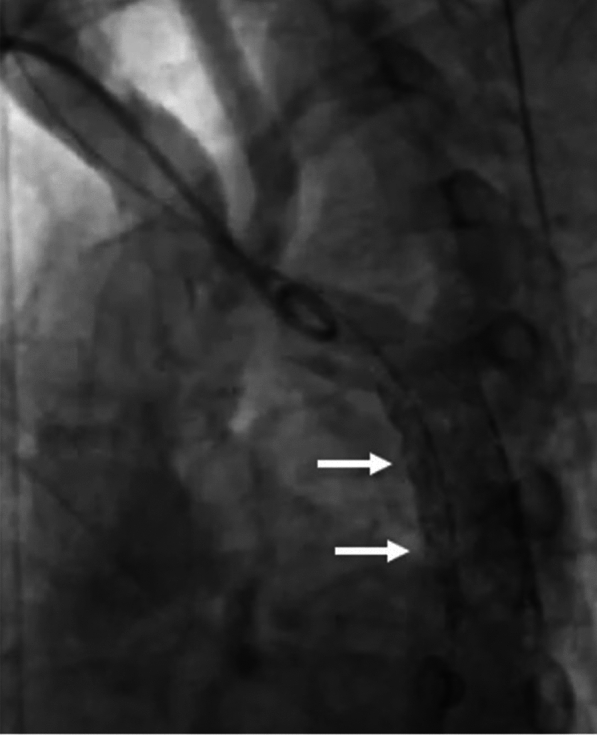
Arch angiogram showing well-opened stent across the CoA segment

After that we pulled down the entire system after inflating another balloon proximally, and this time, we were able to position the stent across the CoA segment. The same balloon was deflated and pulled into the stent, and the balloon stent assembly was inflated after proper positioning. Consequently, the stent was dilated with a larger 16-mm balloon and fully deployed across the CoA segment (Figs. 7, 8)—the gradient decreased to less than 5 mm Hg after stent deployment.

**Fig. 9:**
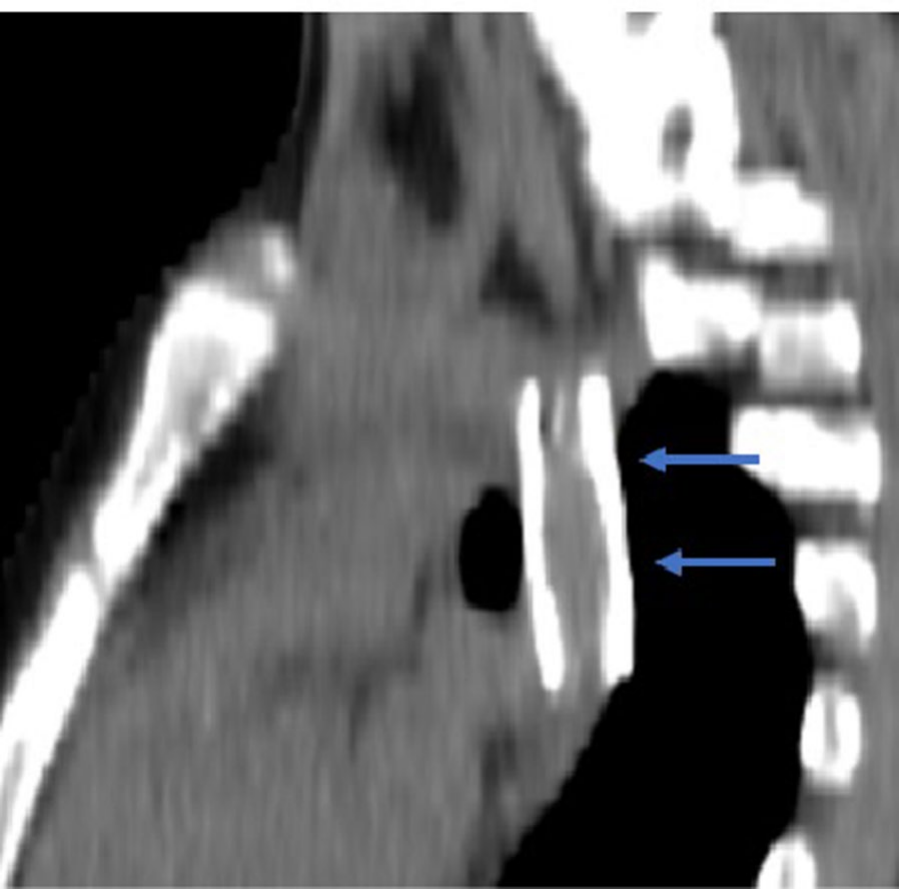
Follow-up CT image at 6 months showing well-expanded stent with no complications

The access site was closed with Perclose ProGlide Suture-Mediated Closure System, and the patient was discharged after two days. A follow-up CT scan showed the stent in situ with no significant narrowing or other complications (Fig. 9).

## Discussion

We describe a rare but potentially life-threatening complication of coarctation of the aorta (CoA) stenting, often requiring prompt management to avoid severe vascular injury or compromised blood flow. Although surgical management is usually warranted in such cases, we describe its successful transcatheter management.

The stent migration can occur proximally or distally, with proximal migration often being more challenging to manage due to the involvement of aortic arch branches. Factors contributing to stent migration include inadequate crimping of the stent on the delivery balloon, improper selection of stent or balloon size, leading to insufficient anchoring at the CoA site, and excessive manipulation of the wire-catheter assembly, which can dislodge a partially deployed stent [2]. In our case, the stent migrated proximally into the right brachiocephalic artery despite hypotensive pacing and appropriate crimping, emphasizing that migration remains an unpredictable risk even with optimal preparation. Due to logistic issues, we used a balloon length that was 4-mm shorter than the stent length, which could have been one of the reasons of stent migration as ideally balloon length should be 4–6-mm longer then stent length.

Proximal stent migration is challenging and generally requires immediate surgical intervention [7]. Our patient was a high-risk surgical candidate with underlying Turner syndrome and chronic ITP. The various strategies have been described in the literature, including snaring the migrated stent [8] or using large delivery sheaths for stabilization. A stepwise approach combining several transcatheter techniques was employed successfully in our case. We initially used a low-profile balloon and inflated it distal to the stent, allowing stabilization and controlled traction of the stent. It minimized further migration and allowed repositioning. Besides, the pigtail catheter, inserted from the right radial artery into the brachiocephalic trunk, prevented further migration and helped stabilize the stent. The tightest portion of the coarctation acted as a mechanical barrier, preventing the stent from being repositioned. By predilating this segment with a larger balloon, we widened the pathway, allowing the stent to be advanced across the CoA site. After properly positioning, inflating the balloon within the stent ensured full deployment and anchoring.

## Conclusions

This case highlights the rare but challenging complication of proximal stent migration during coarctation of the aorta (CoA) stenting and demonstrates the feasibility of managing it successfully with transcatheter techniques. A stepwise strategy involving stent stabilization, low-profile balloon-assisted repositioning, and predilation of tight CoA segments can facilitate successful stent repositioning. As transcatheter management of CoA continues to evolve, our case adds to the existing literature by documenting the occurrence and management of this rare complication.

## Data Availability

No datasets were generated or analyzed during the current study.

## Abbreviations

CoA: Coarctation of the aorta
ITP: Idiopathic thrombocytopenia purpura

## References

[1] Baumgartner H, De Backer J, Babu-Narayan SV, Budts W, Chessa M, Diller GP et al (2021) 2020 ESC Guidelines for the management of adult congenital heart disease. Eur Heart J 42:563–645 32860028 10.1093/eurheartj/ehaa554

[2] Forbes TJ, Garekar S, Amin Z, Zahn EM, Nykanen D, Moore P et al (2007) Procedural results and acute complications in stenting native and recurrent coarctation of the aorta in patients over 4 years of age: a multi-institutional study. Catheter Cardiovasc Interv 70:276–285 17630670 10.1002/ccd.21164

[3] Nikolov D, Grigorova V, Petrov I, Ivanov V (2011) Emergency surgical intervention after unsuccessful percutaneous transluminal angioplasty and stenting of aortic coarctation. Interact Cardiovasc Thorac Surg 13:98–100 21525030 10.1510/icvts.2010.265264

[4] Amirghofran AA, Peiravian F, Borzoee M, Emaminia A, Mollazadeh R (2008) A wandering stent in the ascending aorta. J Cardiovasc Med (Hagerstown) 9:969–970 18695443 10.2459/JCM.0b013e32830214b7

[5] Yaylali YT, Evrengul H, Uludag B (2013) Successful management of a proximally migrated stent in a middle-aged woman with unnoticed native aortic coarctation. Int J Cardiol 168(1):e19-21. 10.1016/j.ijcard.2013.05.025 23711454 10.1016/j.ijcard.2013.05.025

[6] Faim DR, Silva PV, Francisco A, Pires A (2022) Transcatheter management of proximal stent migration in coarctation of the aorta. Ann Pediatr Card 15:222–224 10.4103/apc.apc_191_21 PMC9564396 36246759

[7] Amirghofran AA, Peiravian F, Borzoee M et al (2008) A wandering stent in the ascending aorta. J Cardiovas Med (Hagerstown) 9:969–970 10.2459/JCM.0b013e32830214b7 18695443

[8] Kannan BR, Srinivasan M (2012) Stent migration during transcatheter management of coarctation of aorta. Catheter Cardiovasc Interv 79:408–413 21805571 10.1002/ccd.23231

